# Stress Echocardiography to Detect Exercise Pulmonary Hypertension in Patients With Chronic Thromboembolic Pulmonary Disease

**DOI:** 10.1155/pm/4127338

**Published:** 2026-01-28

**Authors:** Adam Dhayyat, Knut Stavem, Øyvind Jervan, Janne Mykland Hilde, Diyar Rashid, Jostein Gleditsch, Waleed Ghanima, Kjetil Steine

**Affiliations:** ^1^ Department of Cardiology, Østfold Hospital, Kalnes, Norway; ^2^ Institute of Clinical Medicine, University of Oslo, Oslo, Norway, uio.no; ^3^ Department of Pulmonary Medicine, Akershus University Hospital, Lørenskog, Norway, ahus.no; ^4^ Health Services Research Unit, Akershus University Hospital, Lørenskog, Norway, ahus.no; ^5^ Department of Cardiology, Akershus University Hospital, Lørenskog, Norway, ahus.no; ^6^ Department of Radiology, Østfold Hospital, Kalnes, Norway; ^7^ Division of Medicine, Østfold Hospital, Kalnes, Norway; ^8^ Department of Haematology, Oslo University Hospital, Oslo, Norway, oslo-universitetssykehus.no

**Keywords:** chronic thromboembolic pulmonary disease, chronic thromboembolic pulmonary hypertension, exercise pulmonary hypertension, mPAP/CO slope, pulmonary circulation, stress echocardiography

## Abstract

**Background:**

This study was aimed at determining whether stress echocardiography could detect exercise pulmonary hypertension (ePH) in patients with mild chronic thromboembolic pulmonary disease (CTEPD) as compared with right‐heart catheterization (RHC).

**Methods:**

Thirty‐six symptomatic patients with persistent residual perfusion defects detected using ventilation/perfusion scintigraphy underwent a haemodynamic assessment by RHC and echocardiography at rest and during exercise. We compared pulmonary pressures in echocardiography with RHC values using the definitions in current ESC/ERS guidelines for ePH [mean pulmonary artery pressure/cardiac output (mPAP/CO) slope > 3 mmHg/L/min] and PH (mPAP > 20 mmHg).

**Results:**

Ten of the 36 patients (28%) exhibited an increase in the invasive mPAP/CO slope of > 3 mmHg/L/min. The correlation between echocardiographic and invasive measures of the mPAP/CO slope and systolic pulmonary pressure (sPAP) during peak exercise was *ρ* = 0.75 (95*%* *C* *I* = 0.53–0.97) and *ρ* = 0.75 (95*%* *C* *I* = 0.53–0.96), respectively. In bivariate logistic regression analyses, ePH was associated with the echocardiographic sPAP during peak exercise [*o* *d* *d* *s* *r* *a* *t* *i* *o* (*OR*) = 1.13, 95*%* *C* *I* = 1.02–1.24] and with the echocardiographic mPAP/CO slope (*OR* = 3.86, 95*%* *C* *I* = 1.24–12.03). In ROC analysis, AUC was 0.89 (95*%* *C* *I* = 0.78–1.00) for the optimal exercise sPAP cut‐off value of 56 mmHg (*s* *e* *n* *s* *i* *t* *i* *v* *i* *t* *y* = 90*%*, *s* *p* *e* *c* *i* *f* *i* *c* *i* *t* *y* = 87*%*), and 0.84 (95*%* *C* *I* = 0.66–1.00) for the optimal mPAP/CO slope cut‐off value of 3.7 mmHg/L/min (*s* *e* *n* *s* *i* *t* *i* *v* *i* *t* *y* = 89*%*, *s* *p* *e* *c* *i* *f* *i* *c* *i* *t* *y* = 79*%*).

**Conclusion:**

Stress echocardiographic assessments of the exercise sPAP and mPAP/CO slope predicted ePH as measured using RHC with good discrimination and acceptable calibration, providing promising evidence in diagnosing ePH in patients with CTEPD.

**Trial Registration:**

ClinicalTrials.gov identifier: NCT03405480

## 1. Introduction

Chronic thromboembolic pulmonary disease (CTEPD) is characterized by persistent symptoms after pulmonary embolism (PE) due to residual clots in the pulmonary arteries, and more than one in five patients with CTEPD have exercise pulmonary hypertension (ePH), which may contribute to their symptoms [1–4]. ePH is associated with worse clinical outcomes in patients at risk of developing pulmonary hypertension (PH) [5–7]. Right‐heart catheterization (RHC) is the gold standard for assessing pulmonary artery pressures, but it is invasive, expensive and potential harmful, and is usually only performed in specialized centres. In contrast, echocardiography is well tolerated and widely available.

There is scarce information in the literature on the use of stress echocardiography to detect ePH in patients with CTEPD. The exercise systolic pulmonary artery pressure (sPAP) measured using echocardiography is the parameter of choice when ePH is suspected [8–10]. However, this approach does not take into account the linear relationship between pulmonary artery pressure and cardiac output (CO). Therefore, current guidelines define ePH using RHC as a mean pulmonary artery pressure (mPAP)/CO slope of > 3 mmHg/L/min from rest to peak exercise [11], but they provide no recommendation on the possible role of exercise echocardiography such assessments.

This study investigated a population with CTEPD with the main aim of determining whether sPAP or the mPAP/CO slope in exercise echocardiography could be used to detect ePH, defined as an mPAP/CO slope of > 3 mmHg/L/min.

## 2. Material and Methods

### 2.1. Study Design and Recruitment

This was a cross‐sectional study of patients with persistent dyspnoea and residual perfusion defects after PE, and constituted a substudy of the PE‐REHAB project [12].

Patient selection and the RHC data used in this study have been reported previously [13]. Between 1 January 2018 and 1 June 2022, subjects that met the following inclusion criteria were invited by mail to participate in the PE‐REHAB project: (1) PE confirmed (greater than isolated subsegmental emboli) with computed tomography pulmonary angiography (CTPA), (2) age 18–75 years and (3) PE diagnosed 6–72 months prior to inclusion. Patients with the following diagnoses were excluded: (1) heart failure with reduced or preserved left ventricular ejection fraction, (2) moderate or serious valvular heart disease, (3) chronic obstructive pulmonary disease (Global Initiative for Chronic Obstructive Lung Disease stage > 1), (4) restrictive lung disease (total lung capacity < 80% of predicted), (5) CTEPH, (6) pregnancy, (7) active malignancy or (8) any psychiatric or cognitive disorder resulting in failure to comply with the study program. If the diagnostic tests performed in the PE‐REHAB project, which included ventilation/perfusion (V/Q) single‐photon–emission computed tomography pulmonary function tests (spirometry, whole body plethysmography and diffusion capacity of the lungs for carbon monoxide) and resting echocardiography revealed conditions corresponding to the exclusion criteria, these subjects were also excluded.

**Figure 1:**
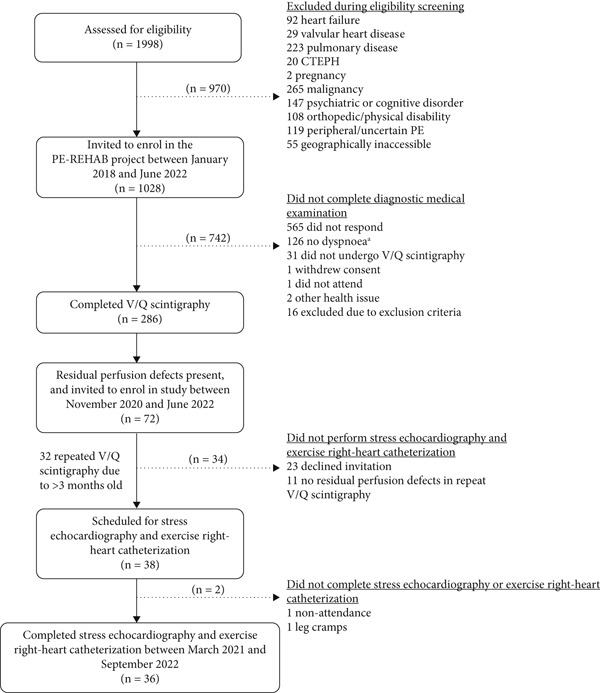
Flow chart of patient selection and attrition.

All patients with persistent perfusion defects detected using V/Q scintigraphy and self‐reported dyspnoea, defined as a modified Medical Research Council dyspnoea grade of ≥ 1 [14], were invited to join this substudy of the PE‐REHAB project. If V/Q scintigraphy had been performed > 3 months previously, it was repeated to ensure the presence of persistent perfusion defects. A flow chart of patient selection and attrition is provided in Figure 1.

The project was approved by the Regional Committee for Medical and Health Research Ethics in Norway (REK no. 2020/20714) and was conducted in accordance with the Declaration of Helsinki. All participants provided written informed consents.

### 2.2. Clinical Evaluations and Ventilation/Perfusion Scintigraphy

We assessed the severity of each participant’s symptoms and their impact on performing the activities of daily living using the World Health Organization functional assessment for PH [11]. V/Q scintigraphic images were acquired using a SPECT/CT (computed tomography) device (GE Discovery NM/CT 670, General Electric Healthcare, Chicago, IL, USA). A specialist in nuclear medicine analysed the images in accordance with the criteria of the European Association of Nuclear Medicine [15], with a positive finding in V/Q scintigraphy defined as a V/Q mismatch in at least one segment or two subsegments of the pulmonary vasculature.

### 2.3. RHC

RHC was performed at rest and during exercise as described previously [13]. We calculated the following haemodynamic variables: pulmonary vascular resistance (PVR) = [mPAP − pulmonary artery wedge pressure (PAWP)]/CO, mPAP/CO slope and PAWP/CO slope. Slopes were defined as multipoint changes (involving at least three data points) from rest to peak exercise, or as two‐point measurements when multipoint measurements were impossible.

The patients were categorized into two groups based on the exercise RHC results: (i) patients with ePH, with mPAP/CO slope > 3 mmHg/L/min; and (ii) no‐ePH patients, with mPAP/CO slope ≤ 3 mmHg/L/min. We further subcategorized the patients as follows for descriptive purposes [11]: (i) no PH at rest, with resting mPAP ≤ 20 mmHg; (ii) mild precapillary PH at rest with resting mPAP > 20 mmHg and < 30 mmHg, PAWP ≤ 15 mmHg and PVR > 2 WU. The post‐capillary contribution during exercise was defined as a PAWP/CO slope of > 2 mmHg/L/min.

### 2.4. Transthoracic Echocardiography

Echocardiography was performed within 3 h before RHC at baseline and during exercise. In 97% (35/36) of cases, echocardiography preceded RHC.

All measurements were performed in accordance with the current recommendations [16]. One investigator (A.D.) performed all resting and exercise echocardiography assessments. All stored images were analysed offline using EchoPAC software (version 204, GE Vingmed Ultrasound, Horten, Norway) by the same investigator. At rest, image acquisition was performed during breath‐holding in the end‐expiratory phase, and three to five consecutive cardiac cycles were recorded. The echocardiographic evaluations included standard measurements made in accordance with current guidelines for the cardiac chambers and function [16] and left ventricle (LV) diastolic function [17].

The evaluations of the right ventricle (RV) systolic function included the tricuspid annular plane systolic excursion (TAPSE) and RV fractional area change (FAC). sPAP was assessed by the tricuspid regurgitation (TR) peak velocity, which was used to estimate the systolic pressure gradient from the RV to the right atrium using the modified Bernoulli equation [4 × (TR jet velocity)^2^]. Echocardiographic estimations of sPAP did not include the right atrial pressure since the latter are rarely available during exercise [18, 19]. The coupling between the right ventricle and pulmonary artery was assessed by calculating the TAPSE‐to‐sPAP ratio (TAPSE/sPAP).

**Figure 2:**
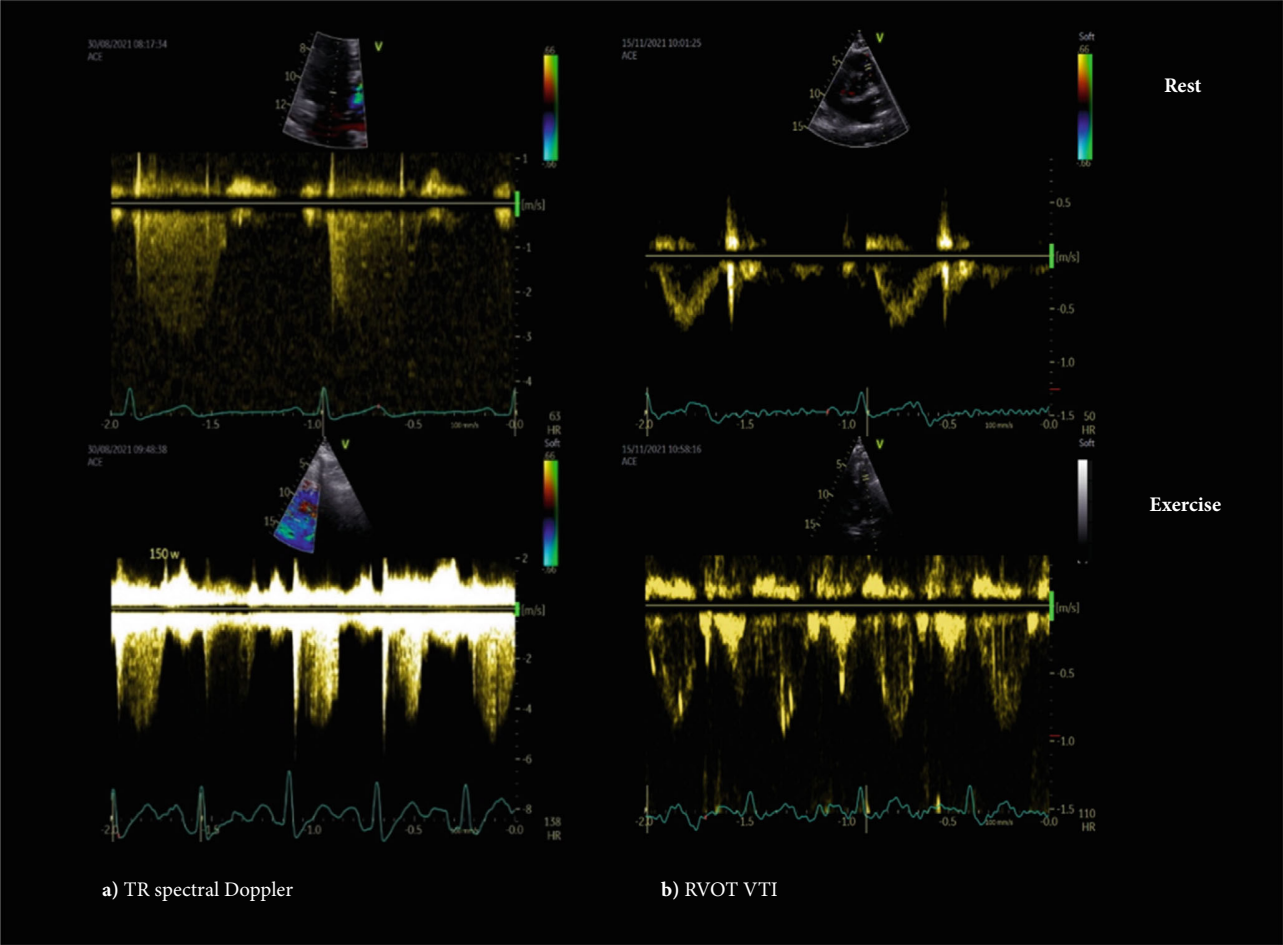
Echocardiographic variables for assessing the mean pulmonary artery pressure/cardiac output (mPAP/CO) slope in two patients. (a) Tricuspid regurgitation (TR) spectral Doppler envelopes demonstrating signal extension throughout systole at rest and with the injection of suspended bubbles in 0.9% NaCl during exercise. (b) Right‐ventricle outflow tract velocity–time integral (RVOT VTI) by Doppler at rest and during exercise.

The TR spectral Doppler envelopes were assessed for quality and were included in the analysis at rest and during exercise only if the signal extended over at least half of systole, or if it had a well‐defined border (Figure 2). mPAP was calculated using the Chemla formula (0.61 × sPAP + 2 mmHg) [20]. CO was derived from the Doppler‐estimated stroke volume in the RV outflow tract (RVOT). LV diastolic dysfunction at rest was defined in accordance with recent recommendations [17].

### 2.5. Stress Echocardiography

The echocardiographic examination during exercise was performed using dynamic supine leg exercise (Ergomed 840 L, Siemens, Erlangen, Germany). The exercise table was tilted laterally by approximately 30° to the left, starting with 4 min of unloaded pedalling at 60 revolutions/min, followed by 25‐W increases every 4 min until exhaustion or until the workload reached 150 W, whichever occurred first. Conventional Doppler echocardiographic parameters were measured at rest, after 30 s in each stage and during peak exercise. Different image sets were acquired in each stage, and at least six cardiac cycles were stored for each set during exercise. At least three cardiac cycles were averaged for each measurement. To optimize the detection of TR, suspended bubbles in 0.9% NaCl were injected into a cubital vein during exercise in all patients. mPAP/CO slopes were measured for multiple levels of exercise, with at least three data points defined as multipoint changes, or as two‐point measurements from rest to peak exercise when multipoint measurements were impossible.

Because premature tachycardia can occur even during low‐intensity exercise, transmitral flow and annular tissue velocities could not be measured accurately beyond exercise at 25–50 W due to the fusion of E‐waves and A‐waves in transmitral Doppler echocardiography. Images were acquired during low‐intensity exercise, typically defined by a heart rate of 85–100 bpm to ensure proper separation of E‐waves and A‐waves.

Diastolic function during exercise was considered to be definitely abnormal when all of the following three conditions were met: (i) average E/e ^′^ > 14 or septal E/e ^′^ ratio > 15 during exercise, (ii) TR peak velocity > 2.8 m/s during exercise and septal e ^′^ velocity of < 7 cm/s or if only lateral velocity was acquired, and (iii) lateral e ^′^ < 10 cm/s at baseline. The results were considered normal when the average (or septal) E/e ^′^ ratio was < 10 during exercise and the TR peak velocity was < 2.8 m/s during exercise. In the absence of these results, the test was considered indeterminate [17].

### 2.6. Statistical Analyses

Data are reported as median (interquartile range) or number (percentage) values, since most of the data were not normally distributed. Continuous and categorical data were compared between groups using the Wilcoxon rank‐sum test or Fisher’s exact test, respectively. Both the invasive and echocardiographic mPAP/CO slopes were calculated for each participant using linear regression.

To assess the intrarater reliability of the echocardiographic analyses, we randomly selected recordings from 10 patients which were reviewed again at least 4 weeks later by the first examiner (A.D.). The same echocardiograms were evaluated by an independent physician (Ø.J.) and compared with the findings with those of the first examiner (A.D.) to assess interrater reliability. The analyses were performed without knowledge of the patients’ conditions. For these analyses, we selected echocardiographic variables of pulmonary haemodynamics, LV diastolic function and RV function. We assessed intra‐ and interobserver measurement variabilities using the intraclass correlation coefficient (ICC) with its 95% confidence interval (CI). The intrarater assessments were performed using a two‐way, mixed‐effects model with absolute agreement, while the interrater assessments used a two‐way, random‐effects model with absolute agreement. An ICC of > 0.70 was considered to indicate acceptable reliability.

The relationships between echocardiographic and invasive haemodynamic measurements of sPAP and the mPAP/CO slope were evaluated using Spearman’s rank correlation coefficient (*ρ*) with the bootstrapped 95% CI, and pairwise agreements were assessed using Bland–Altman analysis with the mean bias, standard deviation (SD) and 95% limit of agreement. Correlations were interpreted based on the *ρ* values as follows: 0.00–0.19, very weak; 0.20–0.39, weak; 0.40–0.59, moderate; 0.60–0.79, strong; and 0.80–1.00, very strong [21].

We assessed the associations between the sPAP and mPAP/CO measurements made using echocardiography and ePH (defined as an mPAP/CO slope of > 3 mmHg by RHC, which was considered the gold standard) using bivariate logistic regression models. The discrimination performance of the models was depicted using receiver operating characteristics (ROC) curves and quantified using the areas under the ROC curves (AUCs). We selected the point on the ROC curve closest to the top‐left corner of the plot as the optimal cut‐off value. We assessed the calibration of the models using the Hosmer–Lemeshow chi‐square test and a calibration plot.

We applied a 5% significance cut‐off in two‐sided tests. All analyses were performed using Stata SE software (version 18, StataCorp, College Station, TX, USA).

## 3. Results

### 3.1. Demographic Characteristics and RHC

Based on the RHC results, 10 of the 36 patients (28%) had an mPAP/CO slope of > 3 mmHg/L/min and were classified as ePH. These patients with ePH included four with mild PH. The remaining 26 patients with an mPAP/CO slope of ≤ 3 mmHg/L/min included three with mild PH. One patient with CTEPD with neither ePH nor PH had atrial fibrillation with a normal heart rate at the time of testing.

Demographic characteristics, measurements at enrolment and invasive haemodynamic data at rest and during peak exercise are presented for patients with and without ePH in Tables 1 and 2. Age was higher in those with than without ePH, while the sex, perfusion defect volume, systemic blood pressure and body size distributions did not differ significantly between the groups.

**Table 1:** Patient characteristics and measurements at enrolment.

	**ePH (** **n** = 10**)**	**No ePH (** **n** = 26**)**
Age, years	71 [68–74]	59 [52–67]
Sex, male	5 (50)	15 (58)
Body mass index, kg/m^2^	30 [27–32]	27 [25–29]
Perfusion defect volume, %	6 [4–8]	5 [3–8]
Anticoagulant treatment	10 (100)	21 (81)
Diabetes	3 (30)	2 (8)
Coronary artery disease	0 (0)	2 (8)
History of hypertension	7 (70)	9 (35)
Smoking status		
Current	0 (0)	3 (12)
Former	4 (40)	10 (39)
Never	6 (60)	13 (50)
FEV_1_/FVC	0.72 [0.68–0.77]	0.76 [0.74–0.80]
DLCO/VA, % of predicted	91 [79–105]	92 [81–102]
WHO functional class I/II/III/IV	0/7/3/0	2/20/4/0
mISWT distance, m	585 [470–680]	880 [620–960]
Time interval between tests, h^a^	2 [2–3]	2 [2–2]

*Note:* Data are median [interquartile range] or *n* (%) values.

Abbreviations: CO, cardiac output; DLCO, diffusing capacity of the lungs for carbon monoxide; ePH, exercise pulmonary hypertension; FVC, forced vital capacity; FEV_1_, forced expiratory volume in 1 s; mISWT, modified incremental shuttle walk test; mPAP, mean pulmonary artery pressure; VA, alveolar volume; WHO, World Health Organization.

^a^Time interval between echocardiography and right heart catheterization.

**Table 2:** Right heart catheterization (RHC) data at rest and during peak exercise in ePH and no‐ePH patients.

	**ePH (** **n** = 10**)**	**No ePH (** **n** = 26**)**	**p**
RHC at rest			
mPAP, mmHg	20 [18–23]	17 [13–20]	0.011
PAWP, mmHg	10 [8–12]	8 [6–10]	0.13
RAP, mmHg	4 [2–6]	5 [2–6]	0.51
PVR, WU	2.1 [1.4–2.4]	1.3 [1.1–1.7]	0.030
CO, L/min	5.6 [5.0–6.3]	6.0 [5.0–7.1]	0.58
sBPrest, mmHg	159 [145–164]	150 [138–164]	0.37
dBPrest, mmHg	67 [63–69]	65 [61–73]	0.48
RHC during peak exercise			
mPAP, mmHg	43 [39–49]	31 [27–36]	< 0.001
PAWP, mmHg	21 [19–25]	15 [13–19]	0.004
RAP, mmHg	8 [7–12]	7 [4–9]	0.041
PVR, WU	2.0 [1.8–2.3]	1.1 [0.8–1.4]	< 0.001
CO, L/min	11.8 [9.7–12.7]	14.1 [12.1–16.0]	0.018
mPAP/CO slope, mmHg/L/min	3.9 [3.4–5.0]	1.8 [1.3–2.6]	< 0.001
PAWP/CO slope, mmHg/L/min	2.0 [1.6–3.1]	1.1 [0.4–1.4]	< 0.001
sBPexc, mmHg	209 [189–233]	208 [186–225]	0.74
dBPexc, mmHg	75 [65–81]	71 [66–78]	0.76
Maximum workload, W	75 [50–100]	100 [75–125]	0.031

*Note:* Data are median [interquartile range] values.

Abbreviations: dBP, diastolic blood pressure; exc: exercise; mPAP/CO slope, least‐squares linear regression between mPAP and CO from rest to peak exercise; PAWP, pulmonary artery wedge pressure; PAWP/CO slope, least‐squares linear regression between PAWP and CO from rest to peak exercise; PVR, pulmonary vascular resistance; RAP, right atrial pressure; sBP, systolic blood pressure.

### 3.2. Echocardiographic Characteristics at Rest and During Exercise

All patients had normal RV and LV systolic functions. There was, however, a lower TAPSE/sPAP ratio both at rest and during exercise in the ePH compared with the no‐ePH group. The median sPAP, mPAP and mPAP/CO slope were higher in the ePH group than in the no‐ePH group. Most of the patients had normal LV diastolic function at rest and indeterminate function during exercise (Table 3). TR during peak exercise was successfully assessed in 31 of the 36 patients (86%). Multipoint mPAP/CO slope determination from rest to peak exercise by echocardiography was only possible in 28 of the 36 patients (78%).

**Table 3:** Echocardiographic data at rest and during exercise in ePH and no‐ePH patients.

	**Rest**			**Exercise**		
	**ePH**	**No ePH**	**p**	**ePH**	**No ePH**	**p**
*n*	10	26		10	26	
Heart rate, bpm	70 [61–79]	66 [58–77]	0.93	122 [111–131]	133 [117–146]	0.15
Maximum workload, W				63 [50–100]	100 [75–150]	0.042
TR peak velocity, m/s	2.7 [2.5–3.0]^a^	2.3 [2.0–2.5]^b^	0.005	3.9 [3.7–4.0]	3.4 [2.9–3.6]^b^	< 0.001
sPAP, mmHg	28 [24–35]^a^	21 [17–25]^b^	0.006	62 [56–64]	45 [34–51]^b^	< 0.001
mPAP, mmHg	19 [17–23]^a^	15 [12–17]^b^	0.006	40 [36–41]	29 [23–33]^b^	< 0.001
PVR, WU	2.0 [1.5–2.3]^c^	1.1 [0.9–1.5]^d^	0.004	3.4 [2.9–4.3]^e^	2.3 [1.6–3.7]^b^	0.026
mPAP/CO slope, mmHg/L/min				4.3 [3.8–5.0]^e^	3.1 [2.6–3.6]^h^	0.005
TAPSE, cm	2.3 [2.2–2.6]	2.2 [2.1–2.6]	0.53	2.9 [2.5–3.2]	2.8 [2.7–3.1]^f^	0.99
TAPSE/sPAP	0.9 [0.7–1.0]^a^	1.2 [0.9–1.4]^b^	0.017	0.5 [0.4–0.5]^a^	0.7 [0.5–0.9]^g^	0.007
RV FAC, %	36 [34–40]	38 [35–41]	0.52	45 [40–47]^a^	46 [40–53]^d^	0.23
SV RV, mL	74 [56–80]	69 [60–78]	0.92	77 [69–89]^e^	73 [63–87]^d^	0.68
CO RV, L/min	4.7 [4.0–6.1]	4.8 [4.0–5.5]	0.83	9.2 [7.5–10.2]^e^	9.4 [8.5–11.0]^d^	0.39
LV EDV, mL	115 [93–134]	118 [95–131]	0.86			
RVD1, cm	3.5 [3.4–4.1]	3.6 [3.3–3.8]	0.58			
RAa, cm^2^	16 [14–17]	17 [14–19]	0.20			
LAVI, mL/m^2^	31 [24–38]	29 [24–40]	0.85			
mPAd, cm	2.0 [1.9–2.2]^e^	2.1 [1.8–2.3]	0.73			
LV EF, %	54 [51–56]	57 [52–58]	0.61	55 [52–61]	58 [54–64]^i^	0.31
LV E/A	0.95 [0.89–1.02]	0.90 [0.78–1.06]^f^	0.58	0.97 [0.96–1.15]	0.97 [0.90–1.07]^i^	0.26
LV E/E ^′^ average	10 [9–13]	7 [7–10]	0.009	10 [9–12]	8 [6–9]^f^	0.025
Diastolic function						
Normal	3 (30)	18 (69)		0 (0)	5 (19)	
Indeterminate	6 (60)	8 (31)		9 (90)	21 (81)	
Abnormal	1 (10)	0 (0)		1 (10)	0 (0)	

*Note:* Data are median [interquartile range] or *n* (%) values.

Abbreviations: A4C, apical four chamber view; E, transmitral early diastolic filling peak velocity by pulsed Doppler; E ^′^, average of early diastolic lateral and septal annular velocities of the mitral valve by tissue Doppler; EDV, end‐diastolic volume; EF, ejection fraction; LAVI, left atrium volume index; LV, left ventricle; mPAd, mean pulmonary artery diameter; RAa, right atrial area; RV, right ventricle; RV FAC, RV fractional area change; RVD1, RV basal internal diameter at end of diastole; sPAP, systolic pulmonary artery pressure; TAPSE, tricuspid annular plane systolic excursion; TR, tricuspid regurgitation.

^a^ *n* = 8.

^b^ *n* = 22.

^c^ *n* = 7.

^d^ *n* = 23.

^e^ *n* = 9.

^f^ *n* = 25.

^g^ *n* = 21.

^h^ *n* = 19.

^i^ *n* = 24.

At rest, there was high intrarater agreement for all echocardiographic measures (*I* *C* *C* > 0.90) except TAPSE (*I* *C* *C* = 0.88). During peak exercise, most of ICCs were > 0.90; the exceptions for TAPSE (0.87) and RV FAC (0.82). There was also high interrater agreement between the echocardiographic measures at rest, with ICCs ≥ 0.85. During peak exercise, the interrater ICCs were all ≥ 0.79 (Table S1).

### 3.3. Diagnostic Performances of Stress Echocardiography for Detecting ePH

Exercise echocardiography produced acceptable values for sPAP and the mPAP/CO slope in 32 and 28 patients, respectively. The coefficient for the correlation between echocardiographic and invasive measures of sPAP was *ρ* = 0.65 [95*%* *C* *I* = 0.45–0.85, *b* *i* *a* *s* = 5.4 ± 7.1 *m* *m* *H* *g* (mean ± SD), *n* = 30] at rest and *ρ* = 0.75 (95*%* *C* *I* = 0.53–0.97, *b* *i* *a* *s* = 3.1 ± 9.2 *m* *m* *H* *g*, *n* = 32) during peak exercise (Figure 3a,b). The coefficient for the correlation between echocardiographic and invasive measures of mPAP/CO slope was *ρ* = 0.75 (95*%* *C* *I* = 0.52–0.97, *b* *i* *a* *s* = –0.52 ± 1.6 *m* *m* *H* *g*, *n* = 28) (Figure 3c,d).

Figure 3Comparisons between measurements made using echocardiography and right‐heart catheterization (RHC). (a) Scatter plot of exercise systolic pulmonary artery pressure (sPAP) values obtained using the two methods, with a regression line and Spearman’s *ρ* indicated (*n* = 32). (b) Bland–Altman plot of sPAP measured by echocardiography and RHC (*n* = 32). (c) Scatter plot of mPAP/CO slope obtained using the two methods with a regression line and Spearman’s *ρ* indicated (*n* = 28). (d) Bland–Altman plot of the mPAP/CO slope obtained using the two methods (*n* = 28).

**Figure:**
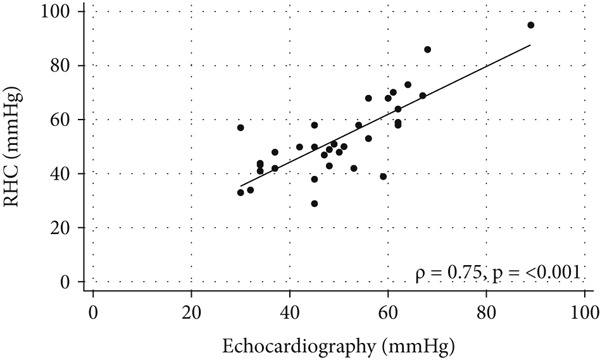
(a)

**Figure:**
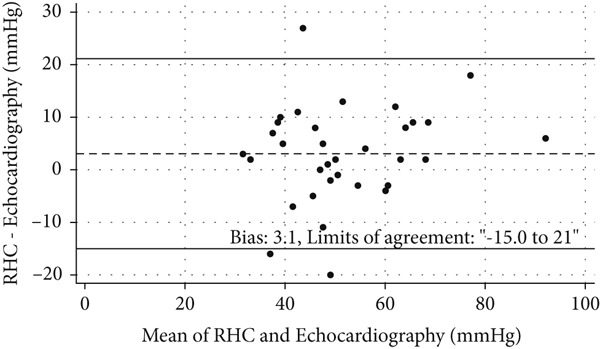
(b)

**Figure:**
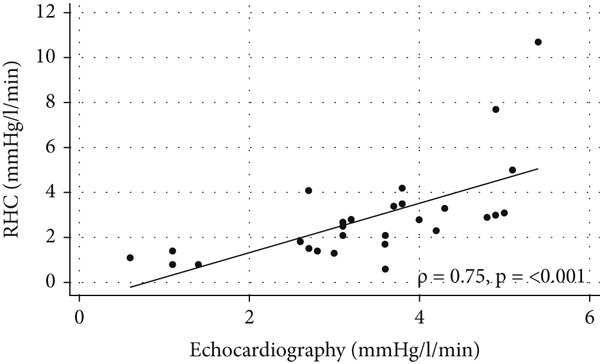
(c)

**Figure:**
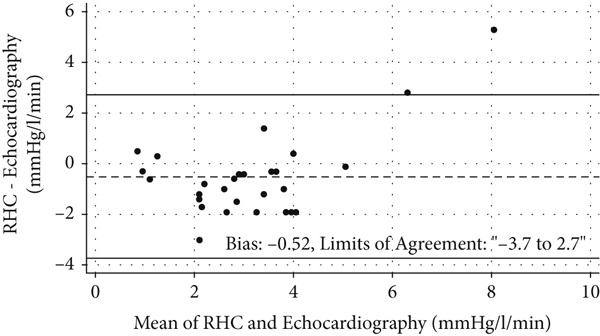
(d)

In bivariate logistic regression analyses, ePH was associated with the echocardiographic sPAP during peak exercise [*o* *d* *d* *s* *r* *a* *t* *i* *o* (*OR*) = 1.13, 95*%* *C* *I* = 1.02–1.24, *n* = 32] and with the echocardiographic mPAP/CO slope (*OR* = 3.86, 95*%* *C* *I* = 1.24–12.03, *n* = 28). Using ePH as the gold standard, the AUC was 0.89 (95*%* *C* *I* = 0.78–1.00) for the optimal exercise sPAP cut‐off value of 56 mmHg (sensitivity and specificity of 90% and 87%, respectively) (Figure 4a) and 0.84 (95*%* *C* *I* = 0.67–1.00) for the optimal mPAP/CO slope cut‐off value of 3.7 mmHg/L/min (sensitivity and specificity of 89% and 79%, respectively) (Figure 4b). The two models demonstrated acceptable calibration (*p* = 0.28 and *p* = 0.50) (Figure 5).

Figure 4ROC curves for using echocardiography variables to predict exercise pulmonary hypertension (ePH) in chronic thromboembolic pulmonary disease: (a) Exercise systolic pulmonary artery pressure (sPAP) (*n* = 32) and (b) mPAP/CO slope (*n* = 28). Filled circle indicates the optimal cut‐off value. AUC, area under the ROC curve.

**Figure:**
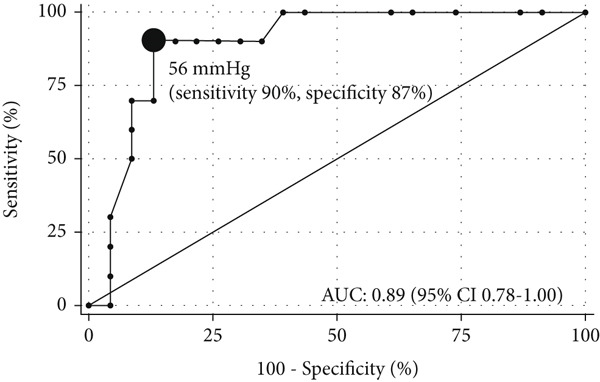
(a)

**Figure:**
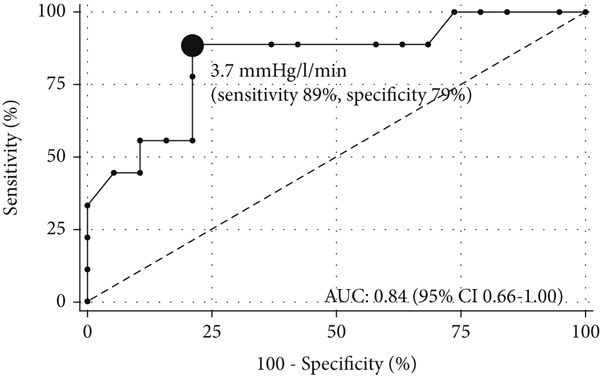
(b)

Figure 5Calibration plots for the predictive model, showing the predicted probability of ePH (*x*‐axis) versus the observed probability (*y*‐axis): (a) exercise sPAP (*n* = 32) and (b) mPAP/CO slope (*n* = 28). Circle size is proportional to the number of patients. Goodness‐of‐fit was quantified using the Hosmer–Lemeshow chi‐square test.

**Figure:**
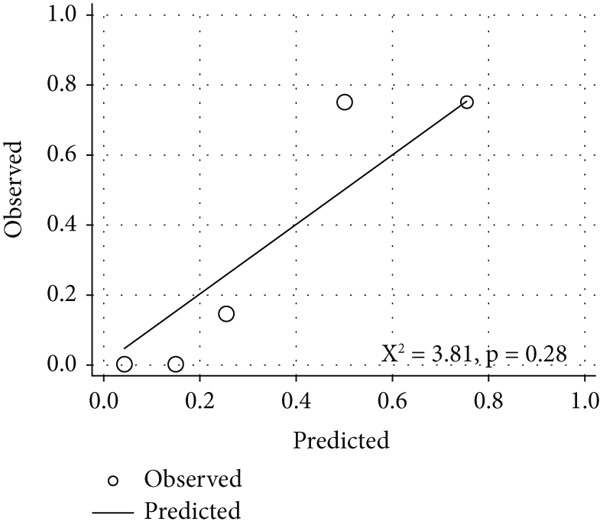
(a)

**Figure:**
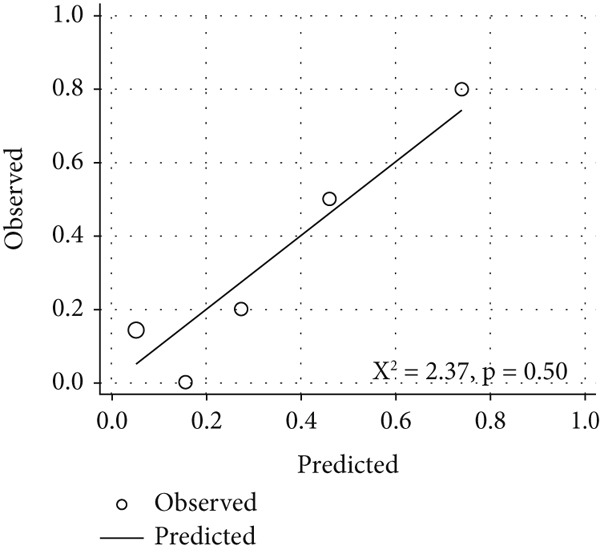
(b)

## 4. Discussion

This study compared the usefulness of stress echocardiography and RHC for detecting ePH in patients with CTEPD using PH and ePH criteria from 2022 [11]. Stress echocardiography was demonstrated to be useful in detecting ePH defined by the current criteria. The results have also demonstrated the feasibility of the assessment of TR, and revealed strong associations between echocardiographic and invasive measures of pulmonary pressures during exercise.

The study used both sPAP and the current criterion of an mPAP/CO slope increase of > 3 mmHg/L/min from baseline to peak exercise to determine the association between stress echocardiographic measurements and those from RHC. During peak exercise, both the echocardiographic sPAP and mPAP/CO slope showed strong correlations with the invasive measurements; however, there were wide limits of agreement. The associations were similar to those found in previous studies comparing echocardiography and RHC in patient populations [19, 22, 23].

Analysis of the TR jet is crucial, and our use of contrast‐enhanced TR signals during exercise contributed to successful measurements in 80% and 86% of patients at rest and during exercise, respectively. We only included the TR jet in the analysis if the signal extended over at least half of systole, or if it had a well‐defined border [22]. It is critical for the TR spectral Doppler envelope to be of high quality since only then will there be a strong correlation between pulmonary pressures measured by stress echocardiography and RHC during exercise, with the correlation only being moderate for reduced‐quality TR Doppler signals [22].

The 10 patients with ePH in this study had an abnormal pulmonary vascular response during exercise that was detectable by echocardiography with a median sPAP of 62 mmHg (*i* *n* *t* *e* *r* *q* *u* *a* *r* *t* *i* *l* *e* *r* *a* *n* *g* *e* = 56–64 *m* *m* *H* *g*). Previous studies have set the upper limit of exercise sPAP at > 50 mmHg, since healthy individuals can reach a maximum sPAP of 40–45 mmHg with a CO of < 20 L/min [8–10]. The optimal cut‐off value in the ROC analysis for an sPAP of 56 mmHg during peak exercise in identifying ePH, defined as an invasive mPAP/CO slope of > 3 mmHg/L/min, was similar to the traditional cut‐off of 50 mmHg used for diagnosing ePH.

Comparing the echocardiographic and invasive mPAP/CO slopes during exercise in a mixed population of healthy subjects and patients with CTEPH or previous PE revealed that echocardiographic determination of the mPAP/CO slope produced similar bias and accuracy values in 87% of the population, but with a lower mPAP/CO slope cut‐off than in the current study [19]. This difference may be attributed to our smaller and different population and the use of a stress echocardiography protocol that allowed our patients to be tested to maximum effort. Our use of the echocardiographic RV CO may also have contributed to the difference, since the geometry of the RVOT is more complex and susceptible to changes in preload and afterload [24]. The mPAP/CO slope in our patients with ePH were higher for echocardiographic than invasive measurements, which may have been due to overestimation of mPAP and underestimation of CO. We also noticed a lower TAPSE/sPAP ratio in ePH, a surrogate marker of right ventricular (RV)‐pulmonary arterial coupling. In our case, the persistently low TAPSE/sPAP ratio both at rest and during exercise in the ePH group, may indicate impaired RV reserve, where the RV fails to sufficiently augment contractility in response to increased afterload. We consider this finding to be supported by the elevated PVR, sPAP and reduced CO observed during exercise RHC [11, 13].

### 4.1. Left‐Ventricle Impact During Exercise

At the group level, our patients with ePH had a median PAWP in the upper part of the normal range during exercise, while the LV diastolic function in echocardiography was mostly normal at rest and indeterminate during exercise. Four of the 10 patients with ePH showed an abnormal PAWP increase, which may therefore have been associated with LV diastolic dysfunction. These findings support previous RHC findings that PAWP increased by > 20 mmHg in 33% of patients with an exercise pulmonary artery pressure increase, even when echocardiography showed no signs of LV systolic or diastolic dysfunction at rest [23].

## 5. Limitations

Our sample was rather small for performing group comparisons and more‐comprehensive analyses. This might have reduced the external validity of the findings, which therefore should be validated in independent and larger cohorts.

Despite confirmatory pulmonary angiography not being performed in the patients with CTEPD, all patients had acute PE as verified by CTPA and a positive finding in V/Q scintigraphy. A negative finding in CTPA would not exclude CTEPD, and our patients did not show any alternative systemic conditions that could explain pulmonary vascular obstruction.

Simultaneously performing echocardiography and RHC would definitely have improved the study, but this was impossible due to technical reasons. Similar to the echocardiographic assessments, RHC was performed in the supine position. Upright positioning would mimic normal physical activity and hence would have been preferable, but this was impossible to achieve in our laboratory. However, the mPAP/CO slope is not influenced by body position [25], and the supine position yields pressure curves that are more stable and reliable. We did not use saline contrast during resting echocardiography, which may have also impacted the obtained results.

## 6. Conclusion

This study has provided insights into the role of stress echocardiography as a diagnostic tool for detecting ePH. Our findings demonstrate the feasibility of using sPAP and the mPAP/CO slope measured echocardiographically during exercise in identifying ePH (as defined by RHC) in patients with CTEPD. However, the present findings need to be validated in independent cohorts.

**Nomenclature**
AUC: area under the receiver operating characteristics curve
CI: confidence interval
CO: cardiac output
CT: computed tomography
CTEPD: chronic thromboembolic pulmonary disease
CTEPH: chronic thromboembolic pulmonary hypertension
ePH: exercise pulmonary hypertension
FAC: fractional area change
ICC: intraclass correlation coefficient
LV: left ventricle
mPAP: mean pulmonary artery pressure
OR: odds ratio
PAWP: pulmonary artery wedge pressure
PE: pulmonary embolism
PH: pulmonary hypertension
PVR: pulmonary vascular resistance
RAP: right atrial pressure
RHC: right‐heart catheterization
ROC: receiver operating characteristics
RV: right ventricle
sPAP: systolic pulmonary artery pressure
SPECT: single‐photon‐emission computed tomography
TAPSE: tricuspid annular plane systolic excursion
TR: tricuspid regurgitation
V/Q: ventilation/perfusion

## Disclosure

All authors have read and approved the final version of the manuscript.

## Conflicts of Interest

Waleed Ghanima received fees for participating in an advisory board from Amgen, Novartis, Pfizer, Principia Biopharma, Sanofi, SOBI, Grifols, UCB, Argenx, Cellphire, Alpine, Kedrion and HiBio; received lecture honoraria from Amgen, Novartis, Pfizer, Bristol Myers Squibb, SOBI, Grifols, Sanofi and Bayer; and received research grants from Bayer, BMS/Pfizer and UCB. The other authors received no commercial support with known competing financial interests or personal relationships that could have influenced the work reported in this manuscript.

## Funding

This study was supported by Sykehuset Østfold and Helse Sør‐Øst RHF.

## Supporting information


**Supporting Information** Additional supporting information can be found online in the Supporting Information section.Table S1The intra‐ and interrater agreement in the interpretation of echocardiographic data, reported as intraclass correlation coefficients with 95% confidence intervals.

## Data Availability

The data that support the findings of this study are available on request from the corresponding author. The data are not publicly available due to privacy or ethical restrictions.

## References

[1] van Kan C. , van der Plas M. N. , Reesink H. J. , van Steenwijk R. P. , Kloek J. J. , Tepaske R. , Bonta P. I. , and Bresser P. , Hemodynamic and Ventilatory Responses During Exercise in Chronic Thromboembolic Disease, The Journal of Thoracic and Cardiovascular Surgery. (2016) 152, no. 3, 763–771, 10.1016/j.jtcvs.2016.05.058, 2-s2.0-84977637125, 27372842. 27372842

[2] Guth S. , Wiedenroth C. B. , Rieth A. , Richter M. J. , Gruenig E. , Ghofrani H. A. , Arlt M. , Liebetrau C. , Prüfer D. , Rolf A. , Hamm C. W. , and Mayer E. , Exercise Right Heart Catheterisation Before and After Pulmonary Endarterectomy in Patients With Chronic Thromboembolic Disease, European Respiratory Journal. (2018) 52, no. 3, 10.1183/13993003.00458-2018, 2-s2.0-85063692461, 30139773. 30139773

[3] Claeys M. , Claessen G. , La Gerche A. , Petit T. , Belge C. , Meyns B. , Bogaert J. , Willems R. , Claus P. , and Delcroix M. , Impaired Cardiac Reserve and Abnormal Vascular Load Limit Exercise Capacity in Chronic Thromboembolic Disease, JACC: Cardiovascular Imaging. (2019) 12, 8 Pt 1, 1444–1456, 10.1016/j.jcmg.2018.07.021, 2-s2.0-85068186607, 30219401. 30219401

[4] Kikuchi H. , Goda A. , Takeuchi K. , Inami T. , Kohno T. , Sakata K. , Soejima K. , and Satoh T. , Exercise Intolerance in Chronic Thromboembolic Pulmonary Hypertension After Pulmonary Angioplasty, European Respiratory Journal. (2020) 56, no. 1, 10.1183/13993003.01982-2019. 32312861

[5] Ho J. E. , Zern E. K. , Lau E. S. , Wooster L. , Bailey C. S. , Cunningham T. , Eisman A. S. , Hardin K. M. , Farrell R. , Sbarbaro J. A. , Schoenike M. W. , Houstis N. E. , Baggish A. L. , Shah R. V. , Nayor M. , Malhotra R. , and Lewis G. D. , Exercise Pulmonary Hypertension Predicts Clinical Outcomes in Patients With Dyspnea on Effort, Journal of the American College of Cardiology. (2020) 75, no. 1, 17–26, 10.1016/j.jacc.2019.10.048, 31918830. 31918830 PMC7043927

[6] Douschan P. , Foris V. , Avian A. , Sassmann T. , Olschewski H. , and Kovacs G. , MPAP/CO-Slope During Exercise as Predictor of Mortality in Patients at Risk for Pulmonary Hypertension, European Respiratory Journal. (2020) 56, no. supplement 64.

[7] Kovacs G. , Bartolome S. , Denton C. P. , Gatzoulis M. A. , Gu S. , Khanna D. , Badesch D. , and Montani D. , Definition, Classification and Diagnosis of Pulmonary Hypertension, The European Respiratory Journal. (2024) 64, no. 4, 2401324, 10.1183/13993003.01324-2024, 39209475. 39209475 PMC11533989

[8] Argiento P. , Vanderpool R. R. , Mulè M. , Russo M. G. , D′Alto M. , Bossone E. , Chesler N. C. , and Naeije R. , Exercise Stress Echocardiography of the Pulmonary Circulation, Chest. (2012) 142, no. 5, 1158–1165, 10.1378/chest.12-0071, 2-s2.0-84865583647. 22539647 PMC3494470

[9] Lim A. Y. , Kim C. , Park S.-J. , Choi J.-O. , Lee S.-C. , and Park S. W. , Clinical Characteristics and Determinants of Exercise-Induced Pulmonary Hypertension in Patients With Preserved Left Ventricular Ejection Fraction, European Heart Journal-Cardiovascular Imaging. (2016) 18, no. 3, 276–283. 10.1093/ehjci/jew199 27679601

[10] Misra D. , Kendes A. , Sulica R. , and Carabello B. , Exercise-Induced Pulmonary Hypertension by Stress Echocardiography: Prevalence and Correlation With Right Heart Hemodynamics, International Journal of Cardiology. (2017) 228, 518–522, 10.1016/j.ijcard.2016.11.191, 2-s2.0-84996533988, 27875728. 27875728

[11] Humbert M. , Kovacs G. , Hoeper M. M. , Badagliacca R. , Berger R. M. F. , Brida M. , Carlsen J. , Coats A. J. S. , Escribano-Subias P. , Ferrari P. , Ferreira D. S. , Ghofrani H. A. , Giannakoulas G. , Kiely D. G. , Mayer E. , Meszaros G. , Nagavci B. , Olsson K. M. , Pepke-Zaba J. , Quint J. K. , Rådegran G. , Simonneau G. , Sitbon O. , Tonia T. , Toshner M. , Vachiery J. L. , Vonk Noordegraaf A. , Delcroix M. , Rosenkranz S. , and Group EESD , ESC/ERS Guidelines for the Diagnosis and Treatment of Pulmonary Hypertension: Developed by the Task Force for the Diagnosis and Treatment of Pulmonary Hypertension of the European Society of Cardiology (ESC) and the European Respiratory Society (ERS). Endorsed by the International Society for Heart and Lung Transplantation (ISHLT) and the European Reference Network on Rare Respiratory Diseases (ERN-LUNG), European Heart Journal. (2022) 43, no. 38, 3618–3731, 10.1093/eurheartj/ehac237. 36017548

[12] Jervan Ø. , Haukeland-Parker S. , Gleditsch J. , Tavoly M. , Klok F. A. , Steine K. , Johannessen H. H. , Spruit M. A. , Atar D. , Holst R. , Dahm A. E. A. , Sirnes P. A. , Stavem K. , and Ghanima W. , The Effects of Exercise Training in Patients With Persistent Dyspnea Following Pulmonary Embolism A Randomized Controlled Trial, Chest. (2023) 164, no. 4, 981–991, 10.1016/j.chest.2023.04.042, 37149257. 37149257

[13] Dhayyat A. , Mykland Hilde J. , Jervan Ø. , Rashid D. , Gleditsch J. , Stavem K. , Ghanima W. , and Steine K. , Exercise Pulmonary Hypertension in Chronic Thromboembolic Pulmonary Disease: A Right Heart Catheterization Study, Pulmonary Circulation. (2024) 14, no. 4, e70018, 10.1002/pul2.70018, 39654659. 39654659 PMC11625648

[14] Fletcher C. , Clifton M. , Fairbairn A. , Fry J. , Gilson J. , Higgins I. , and e a. , Standardized Questionaries on Respiratory Symptoms, British Medical Journal. (1960) 2, no. 5213, 10.1136/bmj.2.5213.1665.

[15] Bajc M. , Neilly J. B. , Miniati M. , Schuemichen C. , Meignan M. , Jonson B. , and EANM Committee , EANM Guidelines for Ventilation/Perfusion Scintigraphy, European Journal of Nuclear Medicine and Molecular Imaging. (2009) 36, no. 8, 1356–1370, 10.1007/s00259-009-1170-5, 2-s2.0-70349238965, 19562336. 19562336

[16] Lang R. M. , Badano L. P. , Mor-Avi V. , Afilalo J. , Armstrong A. , Ernande L. , Flachskampf F. A. , Foster E. , Goldstein S. A. , Kuznetsova T. , Lancellotti P. , Muraru D. , Picard M. H. , Rietzschel E. R. , Rudski L. , Spencer K. T. , Tsang W. , and Voigt J.-U. , Recommendations for Cardiac Chamber Quantification by Echocardiography in Adults: An Update From the American Society of Echocardiography and the European Association of Cardiovascular Imaging, Journal of the American Society of Echocardiography. (2015) 28, no. 1, 1–39.e14, 10.1016/j.echo.2014.10.003, 2-s2.0-84925515752. 25559473

[17] Nagueh S. F. , Smiseth O. A. , Appleton C. P. , Byrd B. F.3rd, Dokainish H. , Edvardsen T. , Flachskampf F. A. , Gillebert T. C. , Klein A. L. , Lancellotti P. , Marino P. , Oh J. K. , Popescu B. A. , and Waggoner A. D. , Recommendations for the Evaluation of Left Ventricular Diastolic Function by Echocardiography: An Update From the American Society of Echocardiography and the European Association of Cardiovascular Imaging, Journal of the American Society of Echocardiography. (2016) 29, no. 4, 277–314, 10.1016/j.echo.2016.01.011, 2-s2.0-84982182159, 27037982. 27037982

[18] Obokata M. , Kane G. C. , Sorimachi H. , Reddy Y. N. V. , Olson T. P. , Egbe A. C. , Melenovsky V. , and Borlaug B. A. , Noninvasive Evaluation of Pulmonary Artery Pressure During Exercise: The Importance of Right Atrial Hypertension, European Respiratory Journal. (2020) 55, no. 2, 10.1183/13993003.01617-2019, 31771997. PMC13077671 31771997

[19] Claessen G. , La Gerche A. , Voigt J. U. , Dymarkowski S. , Schnell F. , Petit T. , Willems R. , Claus P. , Delcroix M. , and Heidbuchel H. , Accuracy of Echocardiography to Evaluate Pulmonary Vascular and RV Function During Exercise, JACC: Cardiovascular Imaging. (2016) 9, no. 5, 532–543, 10.1016/j.jcmg.2015.06.018, 2-s2.0-84951197329, 26508387. 26508387

[20] Chemla D. , Castelain V. , Humbert M. , Hébert J. L. , Simonneau G. , Lecarpentier Y. , and Hervé P. , New Formula for Predicting Mean Pulmonary Artery Pressure Using Systolic Pulmonary Artery Pressure, Chest. (2004) 126, no. 4, 1313–1317, 10.1378/chest.126.4.1313, 2-s2.0-6344280687, 15486398. 15486398

[21] Schober P. , Boer C. , and Schwarte L. A. , Correlation Coefficients: Appropriate Use and Interpretation, Anesthesia & Analgesia. (2018) 126, no. 5, 1763–1768, 10.1213/ANE.0000000000002864, 2-s2.0-85048115048. 29481436

[22] ACMJv R. , Opotowsky A. R. , Santos M. , Rivero J. M. , Dhimitri A. , BJM M. , Bouma B. J. , Landzberg M. J. , Waxman A. B. , Systrom D. M. , and Shah A. M. , Accuracy of Echocardiography to Estimate Pulmonary Artery Pressures With Exercise, Circulation Cardiovascular Imaging. (2017) 10, no. 4, e005711, 10.1161/CIRCIMAGING.116.005711, 2-s2.0-85017618720, 28360262. 28360262 PMC5408510

[23] Kovacs G. , Maier R. , Aberer E. , Brodmann M. , Scheidl S. , Hesse C. , Troester N. , Salmhofer W. , Stauber R. , Fuerst F. C. , Thonhofer R. , Ofner-Kopeinig P. , Gruenig E. , and Olschewski H. , Assessment of Pulmonary Arterial Pressure During Exercise in Collagen Vascular Disease: Echocardiography vs Right-Sided Heart Catheterization, Chest. (2010) 138, no. 2, 270–278, 10.1378/chest.09-2099, 2-s2.0-77955372468, 20418368. 20418368

[24] Haddad F. , Hunt S. A. , Rosenthal D. N. , and Murphy D. J. , Right Ventricular Function in Cardiovascular Disease Part I Anatomy, Physiology, Aging, and Functional Assessment of the Right Ventricle, Circulation. (2008) 117, no. 11, 1436–1448, 10.1161/CIRCULATIONAHA.107.653576, 2-s2.0-41649103553. 18347220

[25] Kirupaharan P. , Lane J. , Melillo C. , Paul D. , Amoushref A. , Abdi S. A. , and Tonelli A. R. , Impact of Body Position on Hemodynamic Measurements During Exercise: A Tale of Two Bikes, Pulmonary Circulation. (2024) 14, no. 1, e12334, 10.1002/pul2.12334, 38223421. 38223421 PMC10784616

