# Supplementary material for: Stress Echocardiography to Detect Exercise Pulmonary Hypertension in Patients With Chronic Thromboembolic Pulmonary Disease
Source: Pulm Med. 2026 Jan 28;2026:4127338. doi: 10.1155/pm/4127338 (PMC12852060; doi:10.1155/pm/4127338)
Supplement: Supplementary file 1 — Supporting Information Additional supporting information can be found online in the Supporting Information section.Table S1The intra‐ and interrater agreement in the interpretation of echocardiographic data, reported as intraclass correlation coefficients with 95% confidence intervals. [file PM-2026-4127338-s001.docx]

**Table S1.** Intra- and interrater agreement in interpreting the echocardiographic data.

|  | Intrarater agreement (*n*=10) | Interrater agreement (*n*=10) |
| --- | --- | --- |
| *At rest* |  |  |
| TR peak velocity | 0.99 (0.97–0.99) | 0.82 (–0.04 to 0.98) |
| E velocity | 0.99 (0.96–0.99) | 0.93 (0.35–0.99) |
| Septal E' velocity | 0.94 (0.79–0.99) | 0.87 (0.39–0.97) |
| Lateral E' velocity | 0.99 (0.98–0.99) | 0.92 (0.29–0.99) |
| E/E' average | 0.98 (0.92–0.99) | 0.85 (–0.01 to 0.98) |
| RVOT VTI | 0.98 (0.90–0.99) | 0.86 (–0.02 to 0.98) |
| TAPSE | 0.88 (0.58–0.97) | 0.85 (0.13–0.97) |
| RV FAC | 0.97 (0.88–0.99) | 0.85 (0.49–0.96) |
| *During exercise* |  |  |
| TR peak velocity | 0.93 (0.74–0.98) | 0.83 (0.20–0.98) |
| E velocity | 0.99 (0.95–0.99) | 0.97 (0.85–0.99) |
| Septal E' velocity | 0.96 (0.85–0.99) | 0.90 (0.04–0.98) |
| Lateral E' velocity | 0.96 (0.85–0.99) | 0.81 (0.11–0.96) |
| E/E' average | 0.99 (0.98–0.99) | 0.95 (0.51–0.99) |
| TAPSE | 0.87 (0.53–0.97) | 0.87 (0.51–0.97) |
| RVOT VTI | 0.90 (0.57–0.98) | 0.79 (0.21–0.96) |
| RV FAC | 0.82 (0.38–0.96) | 0.80 (0.33–0.96) |

Data are intraclass correlation coefficient (95% confidence interval) values.

Abbreviations: E, transmitral early diastolic filling peak velocity by pulsed Doppler; E′, average of early diastolic lateral and septal annular velocities of the mitral valve by tissue Doppler; RVOT VTI, right-ventricle outflow tract velocity–time integral
